# Metformin Protects Myelin from Degeneration in A Mouse Model of Iysophosphatidylcholine-Induced Demyelination in The Optic Chiasm

**DOI:** 10.22074/cellj.2021.7174

**Published:** 2021-03-01

**Authors:** Saman Esmaeilnejad, Saeed Semnanian, Mohammad Javan

**Affiliations:** 1 Department of Physiology, Faculty of Medical Sciences, Tarbiat Modares University, Tehran, Iran; 2 Department of Brain and Cognitive Sciences, Cell Science Research Center, Royan Institute for Stem Cell Biology and Technology, ACECR, Tehran, Iran

**Keywords:** Demyelination, Metformin, Multiple Sclerosis, Neuroprotection

## Abstract

**Objective:**

Multiple sclerosis (MS) is a demyelinating disease of the central nervous system. The autoimmune pathology and long-term inflammation lead to substantial demyelination. These events lead to a substantial loss of oligodendrocytes (OLs), which in a longer period, results in axonal loss and long-term disabilities. Neural cells protection approaches decelerate or inhibit the disease progress to avoid further disability. Previous studies showed that metformin has beneficial effects against neurodegenerative conditions. In this experimental study, we examined possible protective effects of metformin on toxin-induced myelin destruction in adult mice brains.

**Materials and Methods:**

Lysophosphatidylcholine (LPC) was used to induce demyelination in mice optic chiasm. We examined the extent of demyelination at different time points post LPC injection using myelin staining and evaluated the severity of inflammation. Functional state of optic pathway was evaluated by visual evoked potential (VEP) recording.

**Results:**

Metformin attenuated LPC-induced demyelination (P<0.05) and inflammation (P<0.05) and protected against significant decrease (P<0.05) in functional conductivity of optic tract. These data indicated that metformin administration attenuates the myelin degeneration following LPC injection which led to functional enhancement.

**Conclusion:**

Our findings suggest metformin for combination therapy for patients suffering from the myelin degenerative diseases, especially multiple sclerosis; however, additional mechanistic studies are required.

## Introduction

Multiple sclerosis (MS) is a chronic neuroinflammatory disorder affecting myelin sheaths and axons. This disease is described as a remarkable damage to oligodendrocytes (OLs) and myelin destruction (1). The autoimmune invasion and prolonged inflammation leads to a substantial elimination of myelin. These events cause a substantial loss of OLs and their precursors, which in a longer period, results in axonal loss. The long-term disability seen in MS is mainly because of damages to the axons, which are the consequence of the inflammatory attack and deterioration of the axon that remained demyelinated (2). Although the exact cause(s) of the disease is still unclear, both genetic and environmental factors appear to be involved (3). As yet, therapeutic approaches control the disease and limit the recurrence of autoimmune incursion but chronic inflammation remains; therefore, neuroprotection is being accepted as a therapy that may serve to decelerate or inhibit the disease progression to avoid higher levels of disability (4).

Metformin is a member of biguanide drugs and a widely prescribed medication in the treatment of diabetes mellitus (5). A long history of effectiveness and safety has made this small molecule the most frequently prescribed medication worldwide. In addition to its antidiabetic effects, metformin has been demonstrated to be a therapeutically efficient candidate in several central nervous system (CNS) disorders. A previous study demonstrated the neuroprotective effects of metformin in an Alzheimer’s disease model (6). Moreover, the protective effects of metformin on neural cells against apoptosis was also reported (7). Beneficial effects of metformin in neuroinflammatory diseases (8), brain damage models including spinal cord injury (9) ischemia/reperfusion injury (10), Huntington’s (11) and Parkinson’s disease (12) were also reported. There are several studies demonstrating the ability of metformin to hinder the inflammation process in various diseases such as encephalomyelitis, peritonitis-induced sepsis, rheumatoid arthritis, endotoxin-induced uveitis, etc. (13-15). It was proposed that this compound regulates the T helper 1 cells (Th1), Th17, regulatory T cells (Treg) lymphocytes function; such regulatory activity plays a substantial role in its protective effect (16, 17). Metformin’s antioxidant (18) and anti-inflammatory (19) properties alongside the capacity to repair endothelial dysfunction (20) make this medication suitable for MS therapy. Although the mechanism of action of metformin has not yet been fully determined, but previous works showed that metformin-induced activation of AMPactivated protein kinase (AMPK) pathway is a crucial mechanism that triggers the downstream events (21). These pieces of evidence strengthen the idea that metformin may have protective effects on myelin degeneration in animal models of MS. The aim of this study was to examine the protective effect of metformin in a lysophosphatidylcholine (LPC) -induced mouse model of optic nerve demyelination.

## Materials and Methods

### Animals

For this experimental study, 8 to 10-week-old (20-25 g) C57BL/6 male mice were provided by Pasteur Institute (Iran) and housed in plastic cages in groups of four with free access to water and pellet diet; animals were kept at constant temperature (25 ± 2˚C) with 12 hour light/12 hour dark periods.

All animal experiments were conducted in accordance with international guidelines and approved by The Committee for Ethics in Research, Tarbiat Modares University (IR.TMU.REC.1394.189). All efforts were made to minimize the number of animals used and their suffering.

### Induction of demyelination

**Fig.1:**
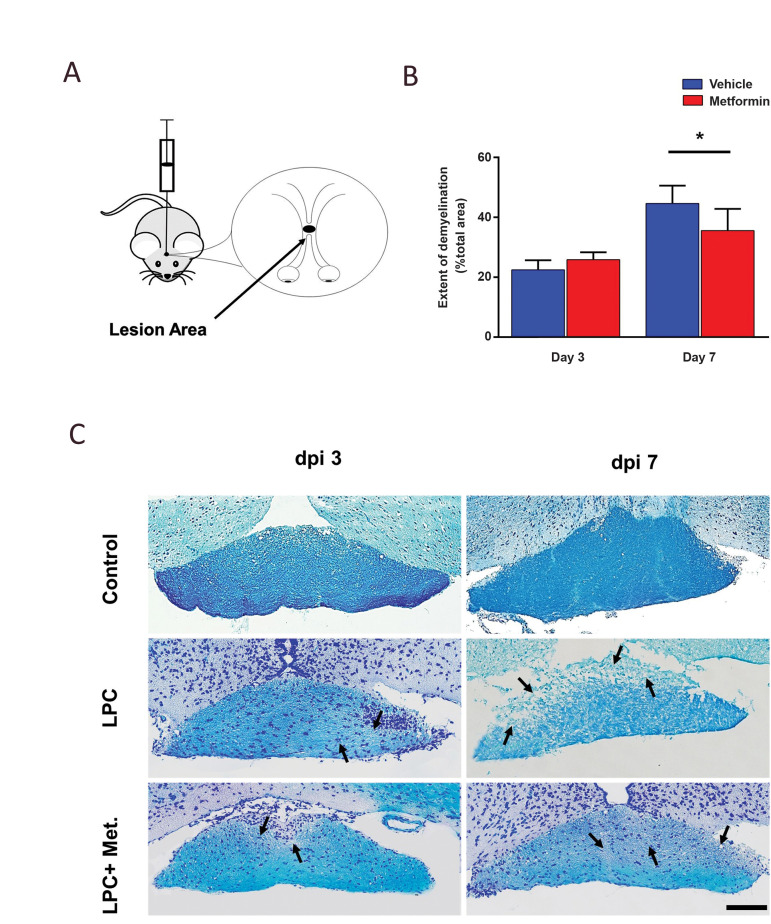
Metformin protects the optic chiasm from demyelination. **A. **Schematic representation of site of injection. **B.** Quantified data for part C. **C.** Representative of LFB-stained micrographs showing the effect of metformin on demyelination in mouse optic chiasm on dpi (days post injection) 3 and 7. The arrows show the demyelinated area (scale bar: 50 µm). Data are shown as mean ± SD (n=3 mice per group). *; P<0.05 shows significant differences compared vehicle, LFB; luxol fast blue, LPC; Lysophosphatidylcholine, and Met; Metformin.

For the surgery, the animals were deeply anesthetized by ketamine [70 mg/kg, intraperitoneal (i.p.); Alfasan, Holland] and xylazine (10 mg/kg, i.p., Alfasan, Holland). Optic chiasm demyelination was performed as mentioned in our previous reports (22) by injecting 1 µl of 1 % LPC (Sigma, St. Louis, USA) dissolved in 0.9% NaCl into the optic chiasm on a stereotaxic apparatus (Fig .1A). The skulls were situated in the stereotaxic apparatus (Stoelting, USA). The coordinates of the injection location were as follows: Anterior: -0.25 mm to the Bregma, lateral: 0, and ventral: 4.9 mm from the Dura (23). LPC was injected into the optic chiasm during 5 minutes. The needle was kept in site for another 5 minutes to avoid reflux through the needle track and was then removed.

### Intervention

Metformin (Merck, Germany) was dissolved in distilled water and daily injected i.p. to the animals. The injection dose of metformin (200 mg/kg) was chosen based on a previous report that showed its effect in a neurodegenerative condition (24). Mice were put into 3 separate groups: i. Control group: animals which received saline, ii. LPC: animals which received local LPC and saline as treatment, and iii. LPC + Met.: animals which received local LPC and metformin for up to 7 days post-lesion (dpi); these groups included subgroups which were sacrificed on days 3 or 7 dpi for immunohistofluorescent studies. 

### Histological analysis

Mice were anesthetized by ketamine and perfused transcardially using phosphate-buffered saline (PBS) and 4% formaldehyde. The brains were harvested and then, 4% buffered formaldehyde was used for post-fixation. The brains were placed in 15% sucrose for one day, and then, transferred to 30% sucrose solution. The brains were molded in optimum cutting temperature (OCT, BioOptica, Italy) compound, then, sectioned by a cryostat apparatus (Histo-Line Laboratories, Italy). Coronal sections of 7 μm thickness containing the optic chiasm, were prepared (23).

For Hematoxylin and Eosin (H&E) staining, the frozen sections were rehydrated in water, immersed in Harris’ Hematoxylin (Bio-Optica, Italy) dye for 4 minutes. The tissues were washed for 3 minutes with tap water, then, placed in acid alcohol and washed again. Eosin staining was performed for 1-2 minutes. The sections were dehydrated by 70, 95 and 100% alcohol concentrations, immersed in xylene and coverslipped by Entellan (Merck Chemicals, Germany). One slide containing 8 sections prepared along the chiasm was stained for each animal. Each group included 3 animals and was evaluated and scored for the severity of inflammation by a pathologist who was blind to the experimental groups. The scores were as follows: 0: no inflammation, 1: a few inflammatory cells, 2: perivascular infiltration, and 3: increased severity of perivascular cuffing extended into the adjacent tissues. The score of inflammation was calculated as the average of its section scores and then, and then groups averages were calculated (25).

For luxol fast blue (LFB) staining, sections were rehydrated in water, immersed in 0.1 % LFB (British Drug House, UK) at 60˚C for 2 hours, placed in 95% alcohol and, then washed under running water each for 10 minutes. The contrast modification was performed by immersion of tissues in 0.05 % lithium carbonate; then, the slides were immersed in water for 10 minutes. The sections were counterstained with 0.1% cresyl violet (Merck, Germany) for 1 minute then, dehydrated in increasing alcohol concentrations. The tissues were cleared in xylene, mounted and then, cover-slipped. ImageJ software was used to measure total and damaged area of the optic chiasm. The extent of demyelination was calculated as the percentage of demyelinated are/total area. The average of the extent of demyelination for each animal was calculated and statistical comparisons were made among the groups. 

For FluoroMyelin (FM) staining, cryosections were incubated with the dye for 20 minutes and 4′,6-diamidino2-phenylindole (DAPI) for another 5 minutes, as stated in the manufacture’s protocol (Molecular Probes, UK). Olympus BX51 fluorescent microscope was used to observe the slides and photography was done using a DP72 camera.

### Quantitative real-time polymerase chain reaction

The optic chiasmata were collected from the mice brains for total RNA isolation using the RiboEx solution (Gene All, Korea) as stated in the manufacturer’s protocol. Reverse transcription and cDNA production were performed by a cDNA reverse transcription Kit (Parstous Biotechnology, Iran) based on the manufacturer’s instructions. The produced cDNA was used for analysis of gene expression. Real-time polymerase chain reaction (q-PCR) was performed by a Real q-PCR Master Mix (Ampliqon, Denmark) on a Rotor-Gene device (Qiagen, Germany). All reactions were performed in duplicate. The relative amount of mRNA was calculated using the delta-delta cycle of threshold (Ct) method, and normalization was done using* Gapdh* as a housekeeping gene. Primer sequences are shown in Table 1.

### Visual evoked potential recording

VEP recording is frequently used for measuring electrical activity of optic pathways in response to a light stimulus. This recording can reflect the extent of demyelination in the optic chiasm region (22). Mice were anesthetized, then, a screw as a recording electrode, was fixed on the surface of occipital cortex of the skull, posterior to Bregma: 3.8 mm, lateral: 3 mm to right. The reference electrode was located on the prefrontal cortex anterior to Bregma: +1, lateral: 1 mm to the left. To tightly fix the electrodes, dental cement was used in the place then, the incision was sutured. Before VEP recording, the mice were maintained for 10 minutes in a dark recording chamber to adapt. For delivering flashing light, an LED light was placed 2 cm away from the left eye. The light was set to flash 150 times at a frequency of 0.5 Hz using a stimulator/ recorder (sampling rate: 10000, bandpass filters: 10 to 100 Hz, gain: 1000X; Science Beam Co., Iran). Responses were averaged and analyzed. The latency of the recorded P1 wave was considered an index of myelination/demyelination of the optic chiasm.

### Statistical analysis

Changes in the extent of demyelinated areas and P1 latency in VEP recordings and FluoroMyelin data were analyzed by Two-tailed unpaired t test. Inflammation scores were analyzed by non-parametric MannWhitney test. P<0.05 were considered statistically significant.

## Results

### Metformin protects the optic chiasm from demyelination 

In order to study the extent of demyelination at dpi 3 and 7, we used LFB and FM staining on frozen sections obtained from the LPC-demyelinated mouse optic chiasmata (26). The assessments done based on LFB staining showed that on dpi 7, in the treated animals, the extent of demyelination was lower than the non-treated animals. The difference between the two groups on dpi 7 was statistically significant (P<0.05, Fig .1B, C). There was no difference between these two groups on dpi 3. 

**Fig.2:**
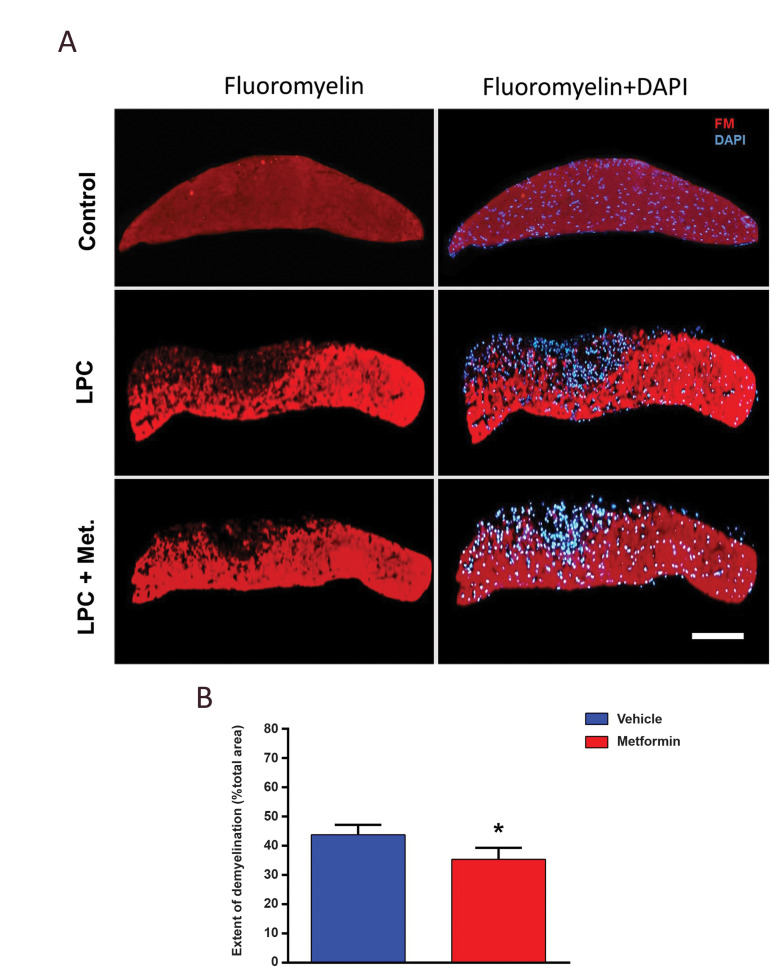
Metformin protects the optic chiasm from demyelination. **A.** Representative micrographs from FM-stained slides showing the effect of metformin on myelin repair in mouse optic chiasm on dpi 7. **B. **Quantified data for the extent of demyelination from FM-stained optic chiasmata (Scale bar: 50 µm). Data are shown as mean ± SD (n=3 mice per group). *; P<0.05 shows significant differences compared to LPC, LPC; Lysophosphatidylcholine, and Met; Metformin.

In order to verify the amount of demyelination, we analyzed the extent of demyelination in micrographs obtained from sections stained with FM. In line with LFB staining results, there was a lower amount of demyelination on dpi 7 in metformin-treated animals compared to non-treated group (Fig .2A, B). These data showed a marked protective effect for metformin against the demyelination process.

**Table 1:** Sequence of primers were used for real-time polymerase chain reaction amplification


Gene	Primers sequence (5ˊ-3ˊ)	Annealing temperature	Product length (bp)

*Mbp*	F: CCCTCAGAGTCCGACGAGCT	62	218
	R: GCACCCCTGTCACCGCTA		
*Gapdh*	F: GGTCGGTGTGAACGGATTTGG	61	198
	R: ATGACAAGCTTCCCATTCTCGG		


### The effects of metformin on inflammation severity following Lysophosphatidylcholine-induced demyelination 

**Fig.3:**
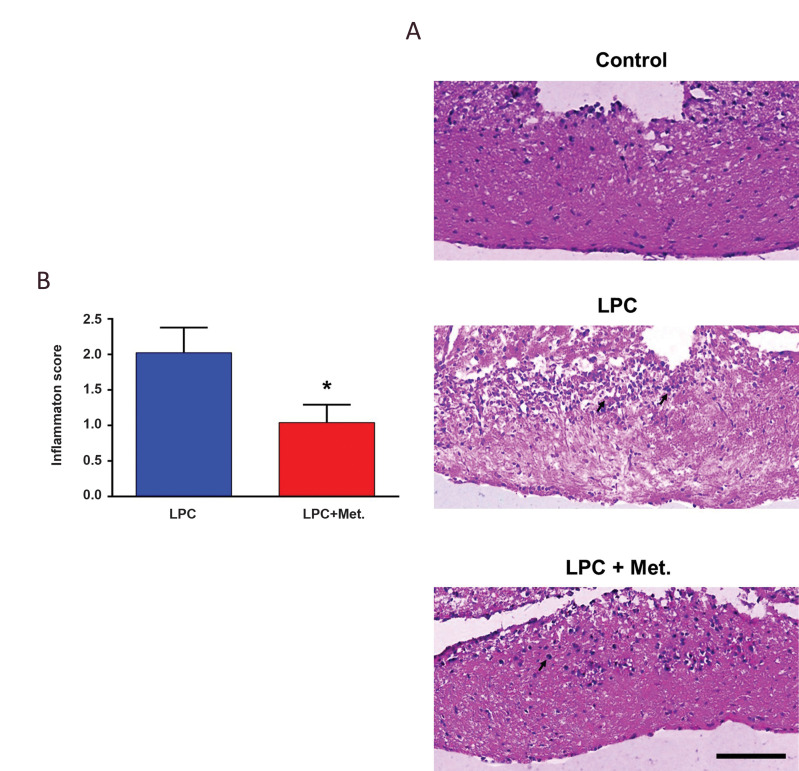
Metformin reduced inflammation in the optic chiasm following LPC-induced demyelination. **A.** Representative micrographs from H&Estained slides showing the effect of metformin on inflammation in mouse optic chiasm on 3 dpi. **B.** Quantitative analysis of H&E-stained sections comparing the inflammation scores for LPC+Met and LPC groups on dpi 3. The arrows show the inflammatory cells (Scale bar: 50 µm). Data are shown as mean ± SD (n=3 mice per group). *; P<0.05 shows significant differences compared to LPC group, LPC; Lysophosphatidylcholine, and Met; Metformin.

LPC administration causes a significant leakage in the blood-brain barrier (BBB) at the injection site (27) which enables robust infiltration of immune cells to the lesion site. To measure the extent of inflammation induced by LPC, the brain samples were collected on dpi 3 for H&E staining. In line with our previous studies (27, 28), our results showed that LPC caused a substantial inflammatory reaction in the injection site (Fig .3A). Quantitative analysis of micrographs obtained from stained sections by a blind pathologist, showed a significant reduction in inflammation score of optic chiasmata in metformintreated animals (Fig .3B, P<0.05). These results showed that the anti-inflammatory effect of metformin probably plays a positive role in its effect on the extent of demyelination. 

### The effect of metformin on level of gene expression

**Fig.4:**
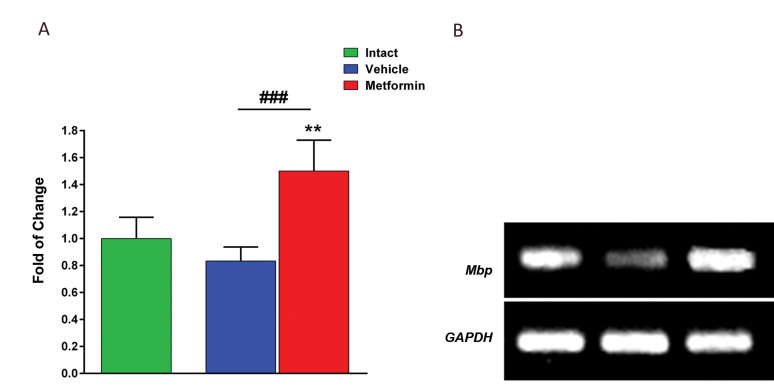
Metformin enhanced level of gene expression. **A.** Changes in the expression of *Mbp* genes within the optic chiasmata following LPC injection on dpi 7 for metformin and non-treated groups. **B.** Representative of *Mbp* and* Gapdh* bands on gel electrophoresis. Data are shown as mean ± SD (n=6 mice per group). **; P<0.01 shows significant differences compared to intact, ###; P<0.01 shows significant differences compared to vehicle, and LPC; Lysophosphatidylcholine.

For further investigation of the molecular basis of these results, we studied the expression of myelinating cell marker, *Mbp*, in treated and non-treated animals on dpi 7 as well as in the control animals. The analysis of gene expression using real-time PCR, showed that the expression of *Mbp* was increased in metformintreated animals in comparison with non-treated animals. These results may indicate that metformin has exerted protective effects on myelinating cells (Fig .4, P<0.01).

### The effects of metformin on the integrity of visual pathway 

**Fig.5:**
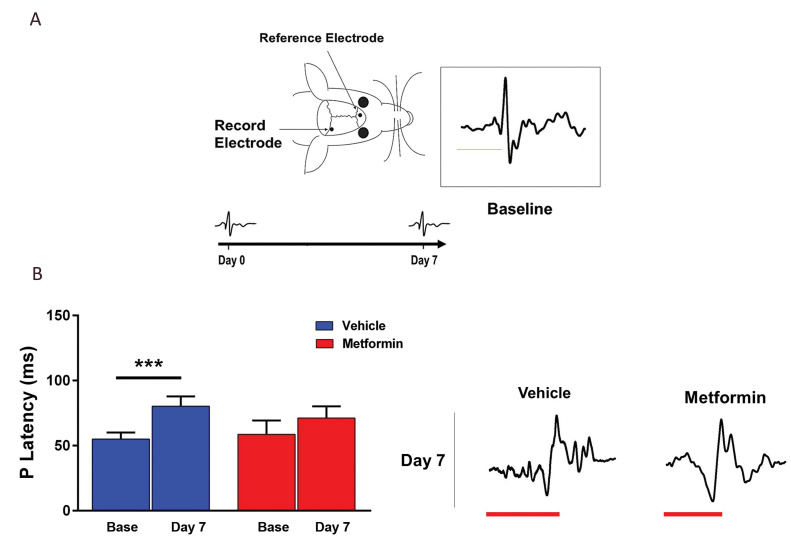
Metformin enhanced recovery of optic tract function after LPC-induced demyelination. **A. **Schematic representation of sites of electrode positioning and experimental procedure for VEP recordings. Box: A sample recording representing the baseline P1-latancy. **B. **Results of quantitative analysis of P1 wave latency at baseline and on 7 dpi. Representative traces for day 7 are mentioned below the graph. Data are shown as mean ± SD (n=5 mice per group). ***; P<0.001 shows significant differences compared to base, LPC; Lysophosphatidylcholine, and VEP; Visual evoked potential.

Visual evoked potential (VEP) recording is a noninvasive approach to assess the functional integrity of optic pathway. While demyelination delays signal conduction, protection restores it to near the normal values. VEPs recorded from the mice visual cortices were used to examine effect of metformin on demyelination of the optic chiasm (22-29). The most stable component of VEP, P1-wave, which was sensitive to LPC-induced optic chiasm demyelination, was selected for further analysis. The recording site and time points of recordings are presented in Figure 5A. The sample VEP recording and P1-latency obtained from a control animal are presented in Figure 5A. Quantitative analysis of P1-wave latency is presented in Figure 5B. Our results shows that, P1 latency was increased on dpi 7 in LPC-injected mice but metformin treatment during days 0-7, reduced the P1 latency time recorded on dpi 7. In fact, metformin administration protected the functional integrity of visual pathway.

## Discussion

Searching for neuroprotective compounds is a major part of developing new treatments for inflammatory and degenerative neurological disorders including MS. One of these medications that have been shown to have beneficial effects in several studies, is metformin. Metformin as the first-line medication for diabetes mellitus, is famous for its few side effects (30, 31). It was shown that metformin exerts many beneficial effects in various pathophysiological conditions. It has anti-oxidative (32), anti-apoptotic (33) and antiinflammatory (13) effects in nervous system diseases. Prolonged metformin therapy decreases the risk of stroke and cardiovascular mortality by 26% (34). After oral administration, this compound crosses the BBB and activates AMPK pathway in the brain cells (11) which plays a fundamental role in cellular processes. There are several studies demonstrating the protective effects of metformin on neuronal cells in a variety of CNS diseases including Parkinson’s and Alzheimer’s diseases (21)(Chiang, 2016 #27;Wang, 2016 #28;Bayliss, 2016 #29;Inzucchi, 2014 #63). Taken the results of all the above-mentioned studies together, it was hypothesized that metformin may have positive effects on the pathology of MS.

In this study, we examined the protective effects of metformin on optic chiasm myelination. Our findings showed that metformin had a protective effect against optic chiasm demyelination induced by LPC. One of the major cellular components triggered by metformin is AMPK. AMPK plays a key role as a master regulator of cellular energy homeostasis. Therefore, this protection may be resulted from its effect on mitochondrial functions as it is widely reported as one of the metformin’s mechanism of action in cell protection. It was also reported that metformin has protective effect on neural and oligodendroglial cells in animal models of cerebral ischemic injury through action on mitochondrial dysfunction which usually occur under neurodegenerative conditions (11, 12).

Neuroinflammation is an essential immune response which includes cellular and vascular events which play a crucial role in escaping the damaging circumstance and controlling the disrupted homeostasis (35). Acute inflammation is a short-term occurrence which is associated with local blood flow increase, elevated permeability in vascular system, immune cell influx, fluid leakage, increased release of cytokines and free radicals (36, 37). In view of MS as an autoimmune disorder mediated by the Th1 and Th17 immune cells, inhibition of the activity of these immune cells can down-modulate the pro-inflammatory immune response and avoid inflammatory response-mediated CNS impairments (38). In this study, we showed that metformin could decrease the inflammatory response after induction of demyelination by LPC administration to the optic chiasm. Our findings are in line with previous studies which showed that metformin can modulate the immune response via decreasing the activity of invading cells and at the same time, increasing the activity of regulatory cells which limits the extent of damage (16, 17).

The results of the molecular investigations showed a significant change in the *Mbp* expression in the injured chiasmata on dpi 7. In an intact tissue, myelinating cells are constantly producing mRNA for Mbp protein maintenance and if there is a reduction in the number of these cells, lower levels of *Mbp* mRNAs will be observed. Our findings show that there was a reduction in *Mbp* expression in non-treated group while an increase in metformin-treated group could be seen. Increased *Mbp* expression in metformin group could be partly due to the greater number of protected myelinating cells and in part, it may be related to the fact that newly formed OLs in the area express higher levels of *Mbp* gene for new myelin sheath production.

We next focused on the functional aspects of the effect of metformin on demyelinated optic chiasm. Our findings showed that metformin prevented functional impairments in the optic pathway after LPC injection to the optic chiasm as the P1 wave latency was preserved from increasing to the higher levels. Our results showed that metformin could attenuate the impairment in the mice visual pathway which was in accordance with the results of our histological analysis. Functional effects of metformin in the context of neural degeneration were studied previously, where this compound improved the memory function in the hypoxic/ischemic brain injured mice, which is in line with our results (24, 39). 

In this study, several limitations must be taken into account. First of all, although we found the beneficial effects of metformin on decreasing the extent of demyelination, the precise mechanism(s) remains to be investigated. Second, it is also remained unclear whether the observed effects are just due to the protective effect of metformin on myelin sheets or it is partially the result of accelerating the remyelination process. Therefore, according to the limitations of our study, we propose conducting further research on the actual mechanism of the observed effect. Despite the promising results of our study, it would be very important to conduct further studies in MS patients by carrying out clinical trials focused on protective capabilities of metformin as an FDA-approved drug.

## Conclusion

In this study, the neuroprotective effects of metformin on mice optic chiasmata damaged by LPC were examined. Our findings showed that metformin inhibited inflammation and protected myelin sheets and significantly preserved the functionality of optic tract as demonstrated by histological, molecular and functional assessments. These results may contribute to finding new therapeutic approaches for multiple sclerosis.

## References

[1] Patrikios P, Stadelmann C, Kutzelnigg A, Rauschka H, Schmidbauer M, Laursen H (2006). Remyelination is extensive in a subset of multiple sclerosis patients. Brain.

[2] Correale J, Marrodan M, Ysrraelit MC (2019). Mechanisms of neurodegeneration and axonal dysfunction in progressive multiple sclerosis. Biomedicines.

[3] Franklin RJ, Ffrench-Constant C (2008). Remyelination in the CNS: from biology to therapy. Nat Rev Neurosci.

[4] Harlow DE, Honce JM, Miravalle AA (2015). Remyelination Therapy in multiple sclerosis. Front Neurol.

[5] Chakraborty A, Chowdhury S, Bhattacharyya M (2011). Effect of metformin on oxidative stress, nitrosative stress and inflammatory biomarkers in type 2 diabetes patients. Diabetes Res Clin Pract.

[6] Asadbegi M, Yaghmaei P, Salehi I, Ebrahim-Habibi A, Komaki A (2016). Neuroprotective effects of metformin against Aβ-mediated inhibition of long-term potentiation in rats fed a high-fat diet. Brain Res Bull.

[7] El-Mir MY, Detaille D, R-Villanueva G, Delgado-Esteban M, Guigas B, Attia S (2008). Neuroprotective role of antidiabetic drug metformin against apoptotic cell death in primary cortical neurons. J Mol Neurosci.

[8] Labuzek K, Liber S, Gabryel B, Okopien B (2010). Metformin has adenosine-monophosphate activated protein kinase (AMPK)- independent effects on LPS-stimulated rat primary microglial cultures. Pharmacol Rep.

[9] Wang C, Liu C, Gao K, Zhao H, Zhou Z, Shen Z (2016). Metformin preconditioning provide neuroprotection through enhancement of autophagy and suppression of inflammation and apoptosis after spinal cord injury. Biochem Biophys Res Commun.

[10] Ge XH, Zhu GJ, Geng DQ, Zhang HZ, He JM, Guo AZ (2017). Metformin protects the brain against ischemia/reperfusion injury through PI3K/Akt1/JNK3 signaling pathways in rats. Physiol Behav.

[11] Jin J, Gu H, Anders NM, Ren T, Jiang M, Tao M (2016). Metformin protects cells from mutant huntingtin toxicity through activation of AMPK and modulation of mitochondrial dynamics. Neuromolecular Med.

[12] Bayliss JA, Lemus MB, Santos VV, Deo M, Davies JS, Kemp BE (2016). Metformin prevents nigrostriatal dopamine degeneration independent of AMPK activation in dopamine neurons. PLoS One.

[13] Nath N, Khan M, Paintlia MK, Singh I, Hoda MN, Giri S (2009). Metformin attenuated the autoimmune disease of the central nervous system in animal models of multiple sclerosis. J Immunol.

[14] Kang KY, Kim YK, Yi H, Kim J, Jung HR, Kim IJ (2013). Metformin downregulates Th17 cells differentiation and attenuates murine autoimmune arthritis. Int Immunopharmacol.

[15] Park DW, Jiang S, Tadie JM, Stigler WS, Gao Y, Deshane J (2013). Activation of AMPK enhances neutrophil chemotaxis and bacterial killing. Mol Med.

[16] Sun Y, Tian T, Gao J, Liu X, Hou H, Cao R (2016). Metformin ameliorates the development of experimental autoimmune encephalomyelitis by regulating T helper 17 and regulatory T cells in mice. J Neuroimmunol.

[17] Paintlia AS, Mohan S, Singh I (2013). Combinatorial effect of metformin and lovastatin impedes T-cell autoimmunity and neurodegeneration in experimental autoimmune encephalomyelitis.J Clin Cell Immunol.

[18] Kukidome D, Nishikawa T, Sonoda K, Imoto K, Fujisawa K, Yano M (2006). Activation of AMP-activated protein kinase reduces hyperglycemia-induced mitochondrial reactive oxygen species production and promotes mitochondrial biogenesis in human umbilical vein endothelial cells. Diabetes.

[19] Isoda K, Young JL, Zirlik A, MacFarlane LA, Tsuboi N, Gerdes N (2006). Metformin inhibits proinflammatory responses and nuclear factor-kappaB in human vascular wall cells. Arterioscler Thromb Vasc Biol.

[20] Majithiya JB, Balaraman R (2006). Metformin reduces blood pressure and restores endothelial function in aorta of streptozotocin-induced diabetic rats. Life Sci.

[21] Markowicz-Piasecka M, Sikora J, Szydlowska A, Skupien A, Mikiciuk-Olasik E, Huttunen KM (2017). Metformin - a future therapy for neurodegenerative diseases : theme: drug discovery, development and delivery in alzheimer’s disease guest editor: Davide Brambilla. Pharm Res.

[22] Pourabdolhossein F, Mozafari S, Morvan-Dubois G, MirnajafiZadeh J, Lopez-Juarez A, Pierre-Simons J (2014). Nogo receptor inhibition enhances functional recovery following lysolecithininduced demyelination in mouse optic chiasm. PLoS One.

[23] Franklin K, Paxinos G (2012). Paxinos and Franklin’s the mouse brain in stereotaxic coordinates.

[24] Wang J, Gallagher D, DeVito LM, Cancino GI, Tsui D, He L (2012). Metformin activates an atypical PKC-CBP pathway to promote neurogenesis and enhance spatial memory formation. Cell Stem Cell.

[25] Yang J, Yan Y, Xia Y, Kang T, Li X, Ciric B (2014). Neurotrophin 3 transduction augments remyelinating and immunomodulatory capacity of neural stem cells. Mol Ther.

[26] Dehghan S, Hesaraki M, Soleimani M, Mirnajafi-Zadeh J, Fathollahi Y, Javan M (2016). Oct4 transcription factor in conjunction with valproic acid accelerates myelin repair in demyelinated optic chiasm in mice. Neuroscience.

[27] Seyedsadr MS, Weinmann O, Amorim A, Ineichen BV, Egger M, Mirnajafi-Zadeh J (2019). Inactivation of sphingosine-1-phosphate receptor 2 (S1PR2) decreases demyelination and enhances remyelination in animal models of multiple sclerosis. Neurobiol Dis.

[28] Yazdi A, Baharvand H, Javan M (2015). Enhanced remyelination following lysolecithin-induced demyelination in mice under treatment with fingolimod (FTY720). Neuroscience.

[29] Satarian L, Javan M, Kiani S, Hajikaram M, Mirnajafi-Zadeh J, Baharvand H (2013). Engrafted human induced pluripotent stem cellderived anterior specified neural progenitors protect the rat crushed optic nerve. PLoS One.

[30] Inzucchi SE, Lipska KJ, Mayo H, Bailey CJ, McGuire DK (2014). Metformin in patients with type 2 diabetes and kidney disease: a systematic review. JAMA.

[31] Werner EA, Bell J (1922). CCXIV.—The preparation of methylguanidine, and of ββ-dimethylguanidine by the interaction of dicyanodiamide, and methylammonium and dimethylammonium chlorides respectively. J Chem Soc, Trans.

[32] Ullah I, Ullah N, Naseer MI, Lee HY, Kim MO (2012). Neuroprotection with metformin and thymoquinone against ethanol-induced apoptotic neurodegeneration in prenatal rat cortical neurons. BMC Neurosci.

[33] Alzoubi KH, Khabour OF, Al-Azzam SI, Tashtoush MH, Mhaidat NM (2014). Metformin eased cognitive impairment induced by chronic l-methionine administration: potential role of oxidative stress. Curr Neuropharmacol.

[34] Selvin E, Hirsch AT (2008). Contemporary risk factor control and walking dysfunction in individuals with peripheral arterial disease: NHANES 1999-2004. Atherosclerosis.

[35] Pandey A, Kumar VL (2016). Protective effect of metformin against acute inflammation and oxidative stress in rat. Drug Dev Res.

[36] Medzhitov R (2008). Origin and physiological roles of inflammation. Nature.

[37] Begum R, Sharma M, Pillai KK, Aeri V, Sheliya MA (2015). Inhibitory effect of Careya arborea on inflammatory biomarkers in carrageenan-induced inflammation. Pharm Biol.

[38] Heikamp EB, Patel CH, Collins S, Waickman A, Oh MH, Sun IH (2014). The AGC kinase SGK1 regulates TH1 and TH2 differentiation downstream of the mTORC2 complex. Nat Immunol.

[39] Dadwal P, Mahmud N, Sinai L, Azimi A, Fatt M, Wondisford FE (2015). Activating endogenous neural precursor cells using metformin leads to neural repair and functional recovery in a model of childhood brain injury. Stem Cell Reports.

